# John Locke on Personal Identity**

**DOI:** 10.4103/0973-1229.77443

**Published:** 2011

**Authors:** Namita Nimbalkar

**Affiliations:** * *Head, Department of Philosophy; Director, Gandhian Studies Centre, Birla College, Kalyan, Maharashtra, India.*; ** *Revised and peer reviewed version of a paper presented at an International Seminar on Mind, Brain, and Consciousness, Thane College Campus, Thane, India, January 13-15, 2010.*

**Keywords:** *Personal Identity*, *Consciousness*, *Self*, *Memory*, *Survival after death*

## Abstract

John Locke speaks of personal identity and survival of consciousness after death. A criterion of personal identity through time is given. Such a criterion specifies, insofar as that is possible, the necessary and sufficient conditions for the survival of persons. John Locke holds that personal identity is a matter of psychological continuity. He considered personal identity (or the self) to be founded on consciousness (viz. memory), and not on the substance of either the soul or the body.

## Introduction

The issue of personal identity and its determents has always been of concern for many philosophers. Questions are raised as to what does being the person that you are, from one day to the next, necessarily consist of. Personal identity theory is the philosophical confrontation with the ultimate questions of our own existence, such as who are we, and is there a life after death? This sort of analysis of personal identity provides a set of necessary and sufficient conditions for the identity of the person over time. In the modern philosophy of mind, this concept of personal identity is sometimes referred to as the diachronic problem of personal identity. The synchronic problem is grounded in the question of what features or traits characterise a given person at one time. There are several general theories of this identity problem. In this paper, the views of John Locke and a criticism of his theory of personal identity are presented.

## Against Cartesian Theory

John Locke (29 August 1632-28 October 1704) was one of the philosophers who were against the Cartesian theory that soul accounts for personal identity. Chapter XXVII on “Identity and Diversity” in *An Essay Concerning Human Understanding* (Locke, 1689/1997) has been said to be one of the first modern conceptualisations of consciousness as the repeated self-identification of oneself, in which Locke gives his account of identity and personal identity in the second edition of the Essay. Locke holds that personal identity is a matter of psychological continuity. Arguing against both the Augustinian view of man as originally sinful and the Cartesian position, which holds that man innately knows basic logical propositions, Locke posits an “empty” mind, a *tabula rasa*, which is shaped by experience, and sensations and reflections being the two sources of all our ideas.

Locke creates a third term between the soul and the body, and Locke’s thought may certainly be meditated by those who, following a scientist ideology, would identify too quickly the brain with consciousness. For the brain, as the body and as any substance, may change, while consciousness remains the same. Therefore, personal identity is not in the brain, but in consciousness. However, Locke’s theory also reveals his debt to theology and to Apocalyptic “great day”, which in advance excuses any failings of human justice and therefore humanity’s miserable state. The problem of personal identity is at the centre of discussions about life after death and immortality. In order to exist after death, there has to be a person after death who is the same person as the person who died.

## Consciousness Can Be Transferred from One Soul to Another

Locke holds that consciousness can be transferred from one soul to another and that personal identity goes with consciousness. In section 12 of the chapter “Identity and Diversity”, he raises the question, “…if the same Substance which thinks be changed, it can be the same person, or remaining the same, it can be a different person” (Locke, 1689/1997). Locke’s answer to both of these questions is in the affirmative. Consciousness can be transferred from one substance to another, and thus, while the soul is changed, consciousness remains the same, thereby preserving the personal identity through the change. On the other hand, consciousness can be lost as in utter forgetfulness while the soul or thinking substance remains the same. Under these conditions, there is the same soul but a different person. These affirmations amount to the claim that the same soul or thinking substance is neither necessary nor sufficient for personal identity over time.

Though the distinction between man and person is controversial, Locke’s distinction between the soul or the thing which thinks in us and consciousness is even more radical. One answer is that the distinction solves the problem of the resurrection of the dead. What is this problem? The problem begins with Biblical texts asserting that we will have the same body at the resurrection as we did in this life.

## The Prince and the Cobbler

Locke explicitly tells us that the case of the prince and the cobbler (Feser, 2007, p 66-68) shows us the resolution of the problem of resurrection. The case is one in which the soul of the prince, with all of its princely thoughts, is transferred from the body of the prince to the body of the cobbler, the cobbler’s soul having departed. The result of this exchange is that the prince still considers himself the prince, even though he finds himself in an altogether new body. Locke’s distinction between man and person makes it possible for the same person to show up in a different body at the resurrection and yet still be the same person. Locke focusses on the prince with all his princely thoughts because in his view, it is consciousness which is crucial to the reward and punishment which is to be meted out at the Last Judgment (Uzgalis, 2007). Locke famously called “person” a forensic term, “appropriating actions and their merit; and so belongs only to intelligent agents capable of a law, and happiness, and misery” (Feser, 2007, p70). This means, then, that an account of the identity of persons across time will have forensic - normative - implications. And so it does.

But this interesting border case leads to this problematic thought that since personal identity is based on consciousness, and that only oneself can be aware of his consciousness, exterior human judges may never know if they really are judging - and punishing - the same person, or simply the same body. In other words, Locke argues that you may be judged only for the acts of your body, as this is what is apparent to all but God; however, you are in truth only responsible for the acts for which you are conscious. This forms the basis of the insanity defence: one cannot be held accountable for acts of which one was unconscious - and therefore leads to interesting philosophical questions and criticisms.

## Critics

There are several philosophers who criticised the Lockean memory theory and stated that it was circular and illogical. Joseph Butler accused Locke of a “wonderful mistake”, which is that he failed to recognise that the relation of consciousness presupposes identity, and thus cannot constitute it (Butler, 1736). In other words, I can remember only my own experiences, but it is not my memory of an experience that makes it mine; rather, I remember it only because it’s already mine. So while memory can reveal my identity with some past experiencer, it does not make that experiencer me. What I am remembering, then, insists Butler, are the experiences of a substance, namely, the same substance that constitutes me now.

Thomas Reid was against Locke’s memory theory and tried to reduce it to absurdity (Reid, 1785). He criticised his theories for several reasons. Firstly, Reid believed that personal identity was something that could not be determined by operations, and that personal identity should be determined by something indivisible. Also, he stated that Locke’s main problem was confusing evidence of something with the thing itself. Finally Reid introduced the officer paradox in an attempt to reduce Locke’s Memory theory to absurdity. Suppose that as he was stealing the enemy’s standard (“standard” is the food store or food provisions), a 40-year-old brave officer remembered stealing apples from a neighbour’s orchard when he was 10 years old; and then suppose further that when he was 80 years old, a retired general, he remembered stealing the enemy’s standard as a brave officer but no longer remembered stealing the neighbour’s apples. On Locke’s account, the general would have to be both identical to the apple-stealer (because of the transitivity of the identity relation: he was identical to the brave officer, who himself was identical to the apple-stealer) and not identical to the apple-stealer (given that he had no direct memory of the boy’s experiences).

Another objection is based precisely on the link between identity and ethics: how can identity - sameness - be based on a relation (consciousness) that changes from moment to moment? A person would never remain the same from one moment to the next, “and as the right and justice of reward and punishment are founded on personal identity, no man could be responsible for his actions” (Reid, 1785, p117). But such an implication must be absurd. Also, Butler concurs, expanding the point to include considerations of self-concern.

Both Reid and Butler, then, wind up rejecting Locke’s relational view in favour of a substance-based view of identity (Shoemaker, 2008). What Butler and Reid retain in common with Locke, though, is the belief that identity grounds certain of our patterns of concern, both prudential and moral. As Reid puts it, “Identity… is the foundation of all rights and obligations, and of accountableness, and the notion of it is fixed and precise” (Reid, 1785, p-112). What they disagree over is just what identity consists of. So, if Locke’s view were right, say Reid and Butler, it would require a host of radical changes to our practices of responsibility attribution and prudential deliberation. But, continues the argument, because making such changes would be crazy - we are strongly committed to the correctness of our current ways of doing things - Locke’s view cannot be right. And although Locke disagrees that the implications of his view are crazy, he does agree with the basic methodology. So, while he admits that he has made some suppositions “that will look strange to some readers” (Locke, 1694, p51), he is also at pains to show that our practices are actually already in conformity with the implications of his view, for example, human law emphasizes the necessity of continuous consciousness, “not punishing the mad man for the sober man’s actions, nor the sober man for what the mad man did” (Locke, 1694, p47). And this is a methodological assumption that has been retained by most theorists on identity and ethics since.

Nevertheless, even if this objection to Locke is thwarted, the others remain in force. For one thing, memory does seem to presuppose personal identity, and so cannot constitute a criterion of it. For another, identity is a transitive relation, while memory isn’t, so the latter cannot be a criterion of the former. Finally, there is the obvious worry that identity seems to persist through the loss of memory: it’s hard to believe that I would cease to exist were I to undergo amnesia. It’s for all these reasons that contemporary theorists working in the Lockean tradition have had to make significant changes to the theory to make it a viable contender for the relation between identity and ethics (Shoemaker, 2008).

## Concluding Remarks [see also Figures 1 and 2]

**Figure 1:**
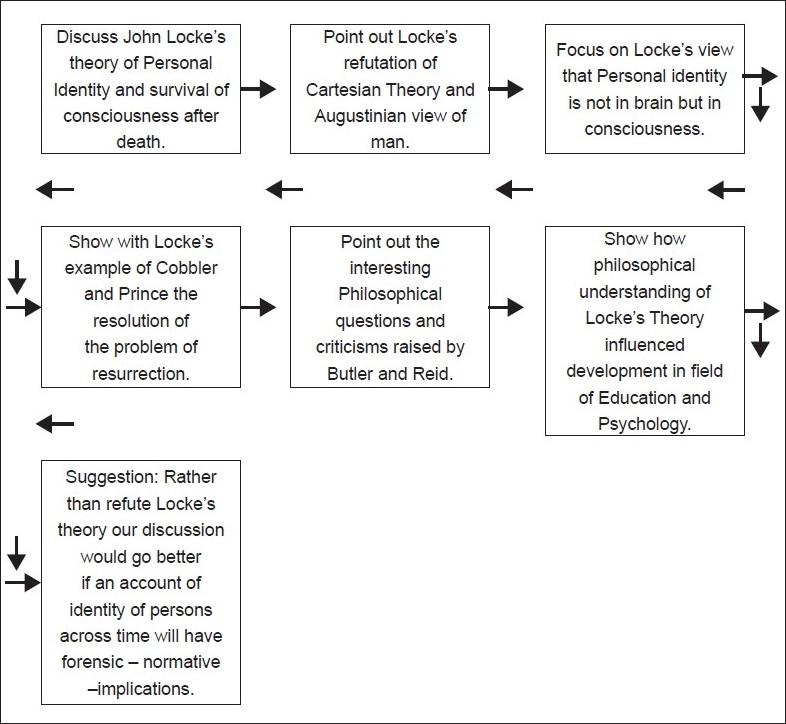
Flowchart of paper - the problem

**Figure 2:**
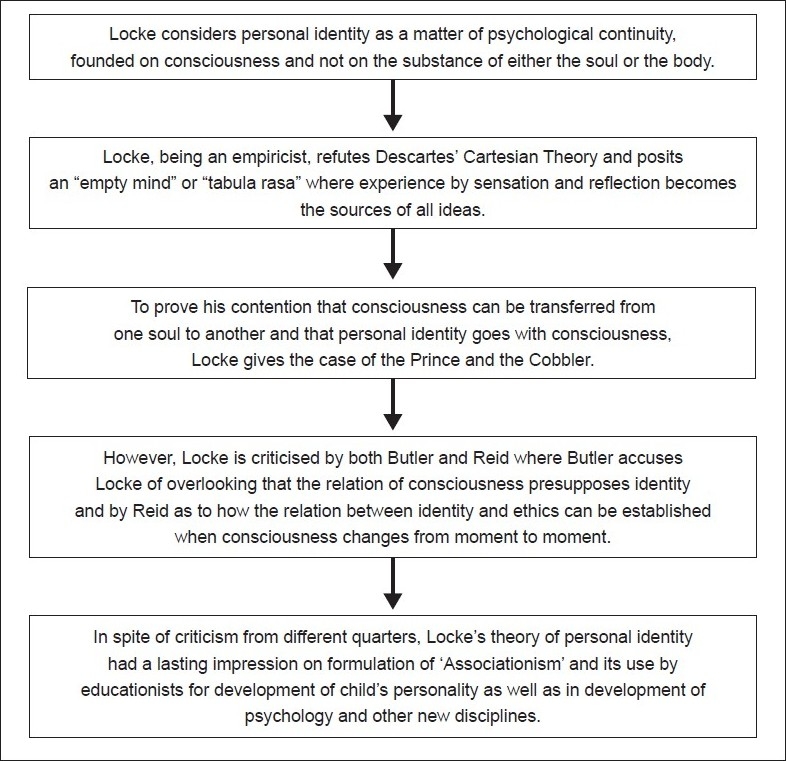
Flowchart of paper - the resolution

Locke’s account of personal identity turned out to be revolutionary. His account of personal identity is embedded in a general account of identity. Locke also wrote, “the little and almost insensible impressions on our tender infancies have very important and lasting consequences” (Locke, 1996, p10). He argued that the “associations of ideas” that one makes when young are more important than those made later because they are the foundation of the self: they are, put differently, what first mark the *tabula rasa*. In his *Essay*, in which is introduced both of these concepts, Locke warns against, for example, letting “a foolish maid” convince a child that “goblins and sprites” are associated with the night for “darkness shall ever afterwards bring with it those frightful ideas, and they shall be so joined, that he can no more bear the one than the other” (Locke, 1689/1997, p357).

“Associationism”, as this theory came to be called, exerted a powerful influence over 18^th^-century thought, particularly educational theory, as nearly every educational writer warned parents not to allow their children to develop negative associations. It also led to the development of psychology and other new disciplines, with David Hartley’s attempt to discover a biological mechanism for associationism in his *Observations on Man* (Hartley, 1749).

### Take Home Message

Personal identity for Locke is psychological continuity. But his theory is criticised by both Butler and Reid as a “wonderful mistake” or “reduced to absurdity”. However, Locke’s theory has had a profound influence in the field of education and the development of psychology.

## Questions That This Paper Raises

1. Apart from memory or consciousness, can any other trait of personal identity persist after the death of an individual?
2. How can a link between identity and ethics be established based on Locke’s model of personal identity?
3. What is the impact of Locke’s theory of identity in the field of education and its implications in formulation of education policy in the current scenario?

## About the Author


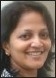
 *Namita A. Nimbalkar, M.A., Ph.D., is an Assistant Professor and Head, Department of Philosophy, and Director, UGC Sponsored Gandhian Studies Centre and Coordinator, Centre for Yoga - Philosophy and Practice at Birla College, Kalyan (M.S.) India. She was awarded Research Fellowship by UGC, New Delhi, and awarded Ph.D. degree in July 2009 by University of Mumbai on “Gandhi’s Concept of Religion and Communal Harmony”. She had been invited as resource person both at National and International level to give talks on Mahatma Gandhi, Peace and Women Empowerment. She has presented a number of research papers at National and International Seminars and Conferences. She has completed two Research Projects and is working on two projects awarded by University of Mumbai and UGC, New Delhi. Under Faculty Exchange Programme, she visited Clayton State University, Atlanta, USA, and in 2010 was invited to deliver a talk on Mahatma Gandhi.*
